# Longitudinal Relationship Between Pain and Depression in People With Inflammatory Arthritis: A Narrative Review

**DOI:** 10.1002/acr.25532

**Published:** 2025-05-08

**Authors:** Natasha Cox, Saeed Farooq, Helen Twohig, Ian C. Scott

**Affiliations:** ^1^ Primary Care Centre Versus Arthritis at Keele University, Keele, Haywood Academic Rheumatology Centre, Haywood Hospital, Midlands Partnership University National Health Service Foundation Trust Staffordshire United Kingdom; ^2^ Primary Care Centre Versus Arthritis at Keele University Keele United Kingdom

## Abstract

As many patients with inflammatory arthritis (IA) have chronic pain, understanding how to best assess and manage pain in IA is a priority. Comorbid depression is prevalent in adults with IA, affecting 15% to 39% of people. Although pain and depression are thought to be associated in IA, this concept is largely based on cross‐sectional data. To better understand potential causality, longitudinal studies are required. This narrative review examines the relationship between pain and depression in patients with IA participating in observational longitudinal studies (spanning association strengths, direction of effect, and causal factors) and summarizes the literature on causal pathways in general populations alongside guideline recommendations/systematic reviews on assessing pain/depression in IA. Fourteen longitudinal studies in IA largely indicate an association between pain and depression, albeit with a small‐to‐modest effect size and a focus on pain intensity. This relationship appears to be bidirectional. Evidence on causal pathways is sparse in IA and limited in non‐IA populations (with structural/function brain changes, altered sensory/pain thresholds, and sleep disturbance implicated) highlighting a need for further research. Although many patient‐reported outcome measures exist to assess pain and depression in IA, evidence for their psychometric properties is often limited, and IA guidelines offer incomplete advice on pain/depression assessment. A simple approach of using a single‐item pain intensity score (eg, a Numeric Rating Scale, which has strong clinimetric properties) in routine IA consultations, with screening for depression where relevant, appears appropriate. Further research is needed to understand how this could be achieved in different health care settings.

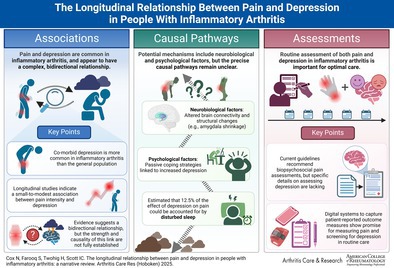

## Introduction

Despite substantial therapeutic advances in treating synovitis, chronic pain remains a problem for many patients with inflammatory arthritis (IA).^1^ Surveys demonstrate that most patients with IA have daily pain of moderate‐to‐severe intensity,^2^, ^3^ and longitudinal studies demonstrate that many patients with rheumatoid arthritis (RA),^4^, ^5^ axial spondylarthritis,^6^ and psoriatic arthritis (PsA) receiving biologic disease‐modifying antirheumatic drugs experience persistent pain.^7^ Chronic pain in IA has widespread impacts, associating with worse quality of life, disability, and fatigue^7^, ^8^, ^9^, ^10^ alongside high levels of long‐term opioid and gabapentinoid prescribing^11^ (despite absent trial evidence of efficacy).^12^ Consequently, understanding how to best assess and manage pain in IA is a priority.^13^

Depression affects an estimated 15% to 39% of adults with IA, depending on the definition used^14^, ^15^ (far higher than the estimated 5% of adults affected in the general population^16^). Comorbid depression in IA has been reported to associate with lesser treatment responses,^17^ worse quality of life,^18^ and higher mortality.^19^ Although widely believed that depression associates with pain in IA, this is largely based on cross‐sectional data showing moderate correlations between depression levels and pain intensity scores.^20^, ^21^, ^22^, ^23^ As longitudinal studies are required to better understand potential causality, the primary aim of this narrative review is to summarize longitudinal observational studies examining the relationship between pain and depression in IA (spanning the strength of associations, direction of effect, and potential causal factors). Its secondary aims are to summarize literature on (a) potential causal pathways underlying the associations between pain and depression in general populations and (b) the best ways to measure and assess pain and depression in routine IA care. This information will be of substantial value to rheumatologists (and other health care professionals, including those in primary care) in informing their use of biopsychosocial IA pain care.

## Methods

To address our primary aim, we searched three bibliographic databases (MEDLINE, PsycINFO, and CINAHL) for longitudinal observational studies examining the relationship between depression and pain in the most common types of IA (RA and/or spondyloarthritis), including studies considering the association between pain and depression and/or variables explaining this relationship. For our secondary aims, we searched PubMed for systematic literature reviews examining (a) causal pathways for the relationship between pain and depression in adults published in the last decade and (b) tools to assess pain/depression in IA. Searches were conducted by one author (NC), with an overview of the approach detailed in Supplementary Figure 1.

## Association between pain and depression in IA

### Overview

This section considers longitudinal study evidence for the association. From 6,505 references, 14 relevant studies were identified (Table 1). All considered pain intensity using a range of analytical methods; two also considered other pain dimensions.

**Table 1:** Longitudinal studies examining the relationship between depression and pain in patients with IA*

Study, year	Region	IA type	Study duration	Size, n	Female, %	Age, years	How the relationship was evaluated	Depression measure	Pain measure	Key findings
Snoeck Henkemans et al,^30^ 2024	The Netherlands	PsA	2 years	367	49	49–51	Association between mean pain and depression scores at any point in study	HADS‐D	VAS	Higher depression scores associated with higher pain scores
Gialouri et al,^26^ 2023	Greece	PsA	29 months	128	52	53	Correlation between changes in depression and pain scores between two clinic visits	HADS‐D	PtP	Weak, statistically significant correlation (r = 0.27, *P* = 0.01)
Rathbun et al,^31^ 2021	USA	RA	2 years	268	11	59–64	Difference in follow‐up pain scores between patients with and without depression at baseline	*ICD‐9*	VAS	Depression associated with higher pain scores at 6 months and 1 year but not at 2 years
Graham‐Engeland et al,^28^ 2016^a^	USA	RA	7 days	31	74	50	Association between baseline depression scores and mean momentary pain scores and the mediating effect of momentary mood on this association	CES‐D	Bespoke	Worse depression scores associated with more intense pain (β = 0.09, *P* < 0.05). Momentary mood did not mediate the pain depression association
Husted et al,^35^ 2012	Canada	PsA	7.5 years	394	29	45	Association between (a) changes in pain and depression scores between visits (and vice versa) and (b) pain scores at previous visit and changes in depression scores between visits (and vice versa)	SF‐36	HAQ	Small bidirectional relationship (standardized regression coefficients < 0.3) between depressive symptoms and pain
McQuillan et al,^33^ 2012	USA	RA	10 years	854	83	58	Association between changes in pain and depression scores	CES‐D	NRS	Changes in pain scores associated with changes in depression scores (β = 0.04; *P* < 0.001)
Odegård et al,^38^	Norway	RA	10 years	149	74	52	Association between pain and depression scores over time (and vice versa)	AIMS	VAS	No association between depression and pain scores (and vice versa)
Conner et al,^34^ 2006^a^	USA	RA	30 days	188	68	56	Association between (a) depression history and mean pain and (b) mean depression and mean pain	DSM‐IV	NRS	Depression history was not associated with pain; current depression associated with pain (β = 7.81, *P* < 0.001)
Chaney et al,^27^ 2004	USA	RA	1 year	42	81	53	Correlation between baseline pain and follow‐up depression scores	IDD	MHAQ‐P	Moderate, statistically significant correlation (r = 0.53; *P* = 0.01)
Smedstad et al,^37^ 1997	Norway	RA	2 years	216	73	52	Direction of effect of association between changes in depression and pain scores over 1 year	AIMS	VAS	Inconsistent results
Ward,^29^ 1994	USA	RA	14 months	24	92	46	Association between changes in depression and pain scores	CES‐D	HAQ	Depression associated with pain (partial R^2^ = 0.06; *P* < 0.0001)
Wolfe and Hawley,^32^ 1993	USA	RA	10 years	713	71	60	Association between changes in pain and depression scores between two clinic visits	AIMS	VAS	Changes in pain scores associated with changes in depression scores
Brown et al,^50^ 1989	USA	RA	6 months	287	75	51	Interaction effects between pain and pain‐coping strategies on depression	CES‐D	AIMS	More use of passive coping and more pain led to more severe depression over time
Brown,^36^ 1990	USA	RA	3.5 years	243	75	53	Association (including direction) over 6 months assessed by causal modeling	CES‐D	VAS	Pain predicted depression in last 6 months; depression did not predict pain
Hawley and Wolfe,^39^ 1988	USA	RA	3.1 years	400^b^	74	55	Association among (a) mean depression and mean pain scores, (b) baseline depression and follow‐up pain scores, and (c) pain and improvement/worsening in depression scores	AIMS	VAS	(a) Mean depression scores associated with mean pain scores, (b) higher baseline depression scores associated with higher follow‐up pain scores, and (c) pain scores did not associate with improvement/worsening in depression scores

* Age is noted as mean, median, or range. AIMS, Arthritis Impact Measurement Scale; CES‐D, Center for Epidemiological Studies Depression; DSM‐IV, *Diagnostic and Statistical Manual, Fourth Edition*; HADS‐D, Hospital Anxiety Depression Scale‐Depression subscale; HAQ, Health Assessment Questionnaire; IA, inflammatory arthritis; *ICD‐9*, *International Classification of Diseases, Ninth Edition*; IDD, Inventory to Diagnose Depression; MHAQ‐P, Modified Health Assessment Questionnaire Pain; NRS, Numeric Rating Scale; PsA, psoriatic arthritis; PtP, Patient Pain Assessment; RA, rheumatoid arthritis; SF‐36, Short Form 36; VAS, Visual Analog Scale.

^a^ Also considered pain appraisal and coping (Conner et al^34^) and pain‐related restrictions (Graham‐Engeland et al^28^).

^b^ Study included mixed population but only findings from patients with IA were considered.

### Correlations between depression and pain

These were reported in two studies. We classified correlation coefficients as very strong (0.8–1), strong (0.6–0.8), moderate (0.4–0.6), weak (0.2–0.4), and very weak (0–0.2).^24^, ^25^ Gialouri et al examined the course of depression and its possible associations with disease‐related variables/patient‐reported outcome measures (PROMs) in 93 patients with PsA enrolled from two hospitals. Data were considered at two sequential visits (between 2019 and 2021). The correlation between changes in depression scores (measured using the Hospital Anxiety Depression Scale [HADS]) and changes in pain intensity scores (measured using a Numeric Rating Scale [NRS]) indicated a weak, statistically significant correlation (r = 0.27, *P* = 0.01).^26^ Chaney et al examined the correlation between baseline pain intensity scores and depression scores at one follow‐up timepoint (mean 12.2 months) in 42 patients with RA. They used the 18‐item Inventory to Diagnose Depression and Modified Stanford Health Assessment Questionnaire Pain scale, reporting a moderate, significant correlation (r = 0.53, *P* = 0.01).^27^

### Depression explaining pain

Four studies evaluated the association between depression as an explanatory variable and pain intensity an outcome variable. Three reported statistically significant associations, and one had mixed findings. First, Graham‐Engeland et al examined whether baseline depressive symptoms predicted mean momentary pain scores in 31 patients with RA over 7 days using multilevel modeling. Depression was measured at baseline using the Centre for Epidemiological Studies Depression Scale (CES‐D) (0–60; ≥19 indicative of depression), and pain was measured five times a day for 7 days (using self‐reported scales, ranging from 0 to 6 for stiffness, pain, and joint tenderness/swelling; the mean of these created a “total pain scale”). Higher baseline depression scores associated with higher pain intensity scores (unstandardized β = 0.09; *P* < 0.05),^28^ indicating that for every 10‐unit increase in baseline depression scores, a corresponding 0.9‐point increase in mean pain scores during follow‐up was observed.

Second, Ward assessed the association between depression and pain in 24 patients with RA over 60 weeks (data collected twice‐weekly) using a pooled time‐series regression. Depression was measured using the CES‐D scale (modified version, excluding items closely related to arthritis to ensure depression was not overestimated) and pain using the Health Assessment Questionnaire (HAQ) pain scale (0–3). Change in depression scores significantly associated with change in pain intensity scores (partial R^2^ = 0.06; *P* < 0.0001), indicating that changes in depression explained 6% of the variation in changes in pain.^29^

Third, Snoeck Henkemans et al examined the association between depressive symptoms and disease activity (including pain) in 367 patients with PsA over 2 years using linear mixed‐effects models. Depression was measured using the depression subscale of HADS (0–21; scores >7 indicating depression) and pain using a 10‐cm Visual Analog Scale (VAS). Depression at any time during follow‐up associated with higher mean pain scores (evidenced by graphical representation, although raw data were not reported).^30^ Fourth, Rathbun et al assessed whether depression affects disease activity (including pain) in 268 veterans with early RA prescribed methotrexate. They used weighted generalized estimating equations to model outcomes at 6, 12, and 24 months. Depression was determined by the presence of an *International Classification of Disease, Ninth Revision* (*ICD‐9*) code, and pain using a 10‐cm VAS. Baseline depression significantly associated with higher pain scores at 6 (*P* = 0.029) and 12 (*P* = 0.028) but not 24 months (*P* = 0.735), suggesting that differences in pain levels by depression status may decline over time. As depression was only measured at baseline, and depression status could have altered over follow‐up, this could have biased 2‐year findings to the null.^31^

### Pain explaining depression

Three studies evaluated the association between pain intensity as an explanatory variable and depression an outcome variable. Two reported statistically significant associations and one had mixed findings. First, Wolfe and Hawley examined the extent to which clinical variables (including pain) explained depression scores between two clinic visits in 713 patients with RA using stepwise regression models. The Arthritis Impact Measurement Scale (AIMS) depression subscale (0–10) measured depression, and a VAS (0–3) measured pain. Change in pain scores significantly associated with change in depression scores (partial R^2^ = 0.04; *P* < 0.001), suggesting that 4% of the change in depression scores could be explained by the change in pain scores.^32^ As most patients had visits <1 year apart, the longer‐term applicability of these findings is uncertain.

Second, McQuillan et al examined associations between changes in RA symptoms (including pain) and depressive symptoms in 854 patients with RA over 10 years (data collected annually) using multilevel modeling. The CES‐D measured depression and an NRS (0–100) measured pain. Changes in pain scores associated with changes in depressive symptoms (unstandardized β = 0.04; *P* < 0.001),^33^ indicating that for every 10‐unit increase in pain scores a 0.4 increase in depression scores was seen.

Third, Conner et al examined the association between depression (as both a binary and continuous variable) and pain intensity in 188 patients over 30 days (data collected using daily diaries). A history of depression was assessed by interview and pain intensity using an NRS (0–100). Using multilevel modeling they reported that, although depression history did not associate with mean daily pain scores, current depression did (unstandardized β = 7.81; *P* < 0.001) indicating that for every additional depressive symptom, a 7.81‐unit increase in pain was observed.^34^

### Bidirectional relationship between pain and depression

Five studies evaluated this using different approaches. One reported statistically significant associations in both directions, two reported mixed findings, and two did not report significant associations. First, Husted et al examined whether depressive symptoms influenced changes in pain scores and vice versa in 394 patients with PsA over 7.5 years using linear mixed‐effects models. Depression was measured using the Short Form 36 (SF‐36) mental component score and pain using the HAQ pain scale. Although the strongest predictors of changes in pain and depressive symptoms between visits were their scores at the previous visit (standardized regression coefficients >0.75) there was evidence of a small bidirectional relationship (standardized regression coefficients <0.3) between depressive symptoms and pain.^35^

Second, Brown examined the extent to which chronic pain and depression were associated in 243 patients with RA, collecting data every 6 months for seven waves (first and final 12 months used for analysis). Depression was measured using the CES‐D and pain using the AIMS pain subscale and 10‐cm pain VAS. Using structural equation modeling, pain scores predicted depression scores over 6 months in the final 12 months (but not the initial 12 months); depression scores did not predict pain scores.^36^

Third, Smedstad et al explored the bidirectional relationship between depression and pain in 216 patients with RA over 24 months. Using multiple linear regression models and a cross‐lagged panel approach they assessed whether changes in depression (AIMS depression scale) influenced subsequent pain levels (100‐mm VAS) and vice versa. Depression scores at one timepoint did not predict pain 1 year later (β = 0.00; *P* = “not significant” at 12 months; β = 0.03; *P* = “not significant” at 24 months). Similarly, pain scores did not predict future depression (β = −0.12; *P* = “not significant” at 12 months; β = 0.06; *P* = “not significant” at 24 months).^37^

Fourth, Odegård et al examined the association between pain and depression in 149 patients with RA over 10 years (follow‐up data collected at 1, 2, 5, and 10 years) using a repeated‐measures mixed model. The AIMS depression scale and 100‐mm pain VAS were used. They observed no significant association between depression and the course of pain and vice versa (statistical values not provided).^38^

Fifth, Hawley and Wolfe, in 400 patients with RA over 4.5 years (data capture every 6 months) used linear regression models to explore the association between mean depression scores and mean pain scores and baseline depression scores and follow‐up pain scores. They also used logistic regression models to explore the association between baseline pain scores and improvements/worsening in depression scores. The AIMS depression and HAQ pain subscales were used. Mean depression scores associated with mean pain scores (β = 0.21; *P* < 0.001)^39^ and higher baseline depression scores associated with higher follow‐up pain scores (β = not reported; *P* = 0.047). Pain did not associate with clinically significant improvements/worsening in depression scores (statistical values not provided).^39^

### Depression and other pain dimensions

Two studies also considered other pain dimensions. Graham‐Engeland et al used multivariable regression modeling to explore the impact of depression at study baseline on mean “pain‐related restrictions” over 1 week (0–6 scale). Patients with depression had higher levels of pain‐related restrictions (unstandardized β = 0.08; *P* < 0.01).^28^ Conner et al used multilevel modeling to assess whether pain “coping strategies” and “coping appraisals” associated with depression.^34^ Pain‐coping strategies were assessed using seven items, with participants asked if they used/thought of various strategies to help with their arthritis pain (eg, relaxation/distraction). Pain‐coping appraisals were assessed using Likert scales and included catastrophizing and positive appraisals. They reported mixed results, with four (of seven) coping strategies (comprising reappraisal, venting emotions, spiritual comfort, and emotional support) and one (of three) coping appraisals (catastrophizing) associating with depression.^34^

### Summary

These longitudinal studies (with a few exceptions) support cross‐sectional studies demonstrating an association between pain and depression in patients with IA. They also suggest a bidirectional relationship; although among the five studies considering the association from both directions there was only a statistically significant bidirectional effect in one study, among the four studies examining depression as an explanatory variable for pain and the three studies examining pain as an explanatory variable for depression, all demonstrated positive and statistically significant associations in some form. This has several caveats. First, the strength of the associations (and corresponding effect sizes) is small to modest. Second, most evidence is for a single dimension (intensity) of a multidimensional symptom (pain). Third, the evidence for this relationship is strongest in short‐ to medium‐duration studies but more conflicting in longer‐term studies.

## Potential causal pathways

### Overview

This section begins by considering pain and depression definitions/mechanisms. It then describes evidence from longitudinal studies examining mediators of the association between pain and depression in IA, followed by systematic literature reviews examining causal pathways in non‐IA populations.

### Pain definition and mechanisms

The International Association for the Study of Pain defines pain as “an unpleasant sensory and emotional experience associated with, or resembling that associated with, actual or potential tissue damage.”^40^, ^41^ This definition was recently updated to include “key notes.” Of particular relevance to depression is the note that pain “is always a personal experience that is influenced to varying degrees by biologic, psychological, and social factors.”^41^ Pain is conceptually classified into three main categories: nociceptive pain (from actual or threatened damage to nonneural tissue, with nociceptor activation), neuropathic pain (from a lesion/disease of the somatosensory nervous system), and nociplastic pain (from altered nociception despite no clear evidence of actual/threatened tissue damage causing peripheral nociceptor activation or evidence for a disease/lesion of the somatosensory system).^42^

Traditionally, pain in IA was thought to be dominated by nociceptor activation from synovial inflammation and joint damage. However, the roles of nociplastic and neuropathic mechanisms are increasingly recognized,^43^, ^44^ with a recent systematic review reporting the prevalence of pain sensitivity/neuropathic‐like pain in patients with IA to be 36% to 42%^45^ and comorbid fibromyalgia estimated to affect 21%, 18%, and 13% of patients with RA, PsA, and ankylosing spondylitis, respectively.^46^ Consequently, it has been proposed that pain in IA is a “mixed type,” defined as “a complex overlap of the different known pain types (nociceptive, neuropathic, [and] nociplastic) in any combination, acting simultaneously and/or concurrently to cause pain in the same body area.”^47^ This complexity complicates IA pain research and treatment.

### Depression definition and mechanisms

Two criteria are often used to define depression: *ICD‐11* and *Diagnostic Statistical Manual of Mental Health Disorders, Fifth Edition*, which use the terms “depressive episode” and “major depressive episode,” respectively. Both require at least five (from nine) characteristic depressive symptoms to be present during the same 2‐week period accompanied by a change from normal functioning.

Depression's underlying mechanisms remain unclear, with various factors considered important, including monoamine deficiency, genetic/environmental factors, neurogenesis, and immunologic/endocrinological abnormalities. Hypothalamic pituitary adrenal axis dysregulation is considered to provide a neurobiological link between many of these factors and depression.^48^ A recent narrative review has examined factors that may underlie the increased prevalence of comorbid depression in IA, with shared cytokine pathways, inflammatory mediated neurologic changes, and the psychological and social impacts of chronic disease considered potentially important.^49^

### Longitudinal studies of causal factors in IA

From 6,505 references, we identified two studies exploring mediators of the association between pain and depression in patients with IA (one was also included in the analysis of associations). First, Brown et al examined the role of coping strategies during pain episodes in predicting depression in 287 patients with RA using cross‐lagged, covariance structural modeling. They conducted two data collection “waves” 2 months apart. They reported that “passive” coping strategies (eg, depending on others/restricting activities) associated with higher levels of depression. Conversely, “active” coping strategies (eg, staying busy/ignoring pain) had lesser associations with depression. They also reported that an interaction between high pain levels and frequent use of passive coping strategies resulted in the greatest levels of depression. Conversely, active coping strategies had an inverse association with depression.^50^

Second, Graham‐Engeland et al, previously described, examined whether baseline depressive symptoms and momentary mood fluctuations (both positive and negative) predicted momentary pain among 31 adults with RA using multilevel modeling. They measured depression at baseline using the CES‐D, with momentary pain and mood data collected five times a day for 1 week. Baseline depressive symptoms associated with higher levels of momentary pain and pain‐related restrictions. Although greater momentary positive mood associated with less pain and fewer pain‐related restrictions, greater momentary negative mood associated with more pain‐related restrictions but did not significantly predict pain after controlling for stress experiences.^28^

### Systematic reviews of causal pathways in non‐IA populations

From 729 potential references, nine relevant systematic reviews were identified (Table 2). Causal pathways considered can be classified as neurobiologic (five reviews), psychosocial (one review), and multiple mechanisms (three reviews).

**Table 2:** Systematic reviews of potential causal pathways for the association between pain and depression in noninflammatory arthritis populations*

Study, year	Aim	Key findings	Limitation(s)
Neurobiologic mechanisms			
Chen et al,^51^ 2023	Examine relationship between emotional stress and amygdala size in patients with chronic pain and emotional stress	Thirteen studies synthesized. Seven indicated significant amygdala shrinkage (measured using MRI) in patients with chronic pain and emotional stress compared with healthy controls (*P* = 0.02)	Not possible to compare amygdala size in those with chronic pain with/without emotional distress because of insufficient studies
Zheng et al,^52^ 2022	Identify neural correlates of comorbid pain and depression, using ALE meta‐analysis	Twenty‐six studies combined in the meta‐analysis. Results indicated that pain with concomitant depression associated with the right amygdala, whereas depression with concomitant pain related to the left dorsolateral prefrontal cortex	Only included studies in meta‐analysis with ALE coordinates corresponding to significant results
Ma et al,^53^ 2022	Collect VBM studies on gray mater volume alterations comparing patients with chronic pain and associated depression to healthy controls to explore brain regions involved in comorbid chronic pain and depression	Eighteen studies included. Compared with healthy controls, people with chronic pain and depression had increased gray matter volume in the left hippocampus (*P* < 0.005) and decreased gray matter volume in the medial part of the left superior frontal gyrus (*P* < 0.005)	The comparison group was healthy controls and people with chronic pain but not depression, or people with depression without chronic pain
Malfliet et al,^54^ 2016	Synthesize literature on the relationship between maladaptive cognitive and emotional factors related to pain and brain alterations in chronic pain using MRI	Fourteen articles considered depressive symptoms. Evidence for the following in relation to depression: (a) inverse relationships with resting state functional connectivity in brain areas involved in pain processing, (b) positive correlation with parts of the default mode network involved in affective‐cognitive processing, and (c) positive association with pain‐induced activation of the left prefrontal cortex	MRI processing varied, making interpretation difficult; unknown if changes also occur in people without chronic pain
Thompson et al,^55^ 2016	Conduct a meta‐analysis to compare depressed and healthy control groups and their response to experimentally induced pain	Thirty‐two studies included in meta‐analysis. Comparing depressed and healthy groups, for high‐intensity noxious stimulation, overall pain tolerance was similar. For low‐intensity stimulation, depressed groups had slightly higher mean sensory threshold and pain threshold	Significant heterogeneity present
Psychosocial mechanisms			
Karimi et al,^56^ 2023	Examine the extent to which sleep disturbance mediates the association between depression and chronic pain	Forty‐nine studies included. A total of 12.5% of the total effect of depression on pain could be explained by the indirect effect of sleep disturbance (moderate certainty in evidence)	Significant heterogeneity was present
Multiple mechanisms			
Antoniou et al,^57^ 2023	Investigate impact of adverse childhood experiences on the neural correlates of chronic pain and depression, using neuroimaging studies	Forty‐nine studies included. Compared with those without adverse childhood experiences, those with depression and adverse childhood experiences had functional and structural brain changes in the superior frontal, lingual gyrus, hippocampus, insula, putamen, superior temporal, inferior temporal gyrus, and anterior cerebellum and those with depression or chronic pain and adverse childhood experiences had changes in the cingulate gyrus, inferior parietal lobule, and precuneus	No studies considered people with both chronic pain and depression
Khan et al,^58^ 2020	Evaluate studies exploring the covariation among pain, depression, and/or anxiety using a twin study design	Six studies examined the covariation between pain and depression. One classic twin study reported shared genetic influences entirely explained the association, three reported significant nonshared environmental effects, one co‐twin control study reported unmeasured genetic and shared environmental effects explained the association, and 1 reported no evidence for unmeasured familial factors	Five of the six studies were cross‐sectional
Dresler et al,^59^ 2019	Explore the association between migraine and psychiatric disorders (including depression)	A total of 178 studies included. For depression, potential neurobiologic mechanisms include shared genes, neurotransmitter systems (serotonin, dopamine, and GABA), shared HPA axis involvement, cytokine imbalances, and neuroplasticity. From a psychologic perspective, stress and personality traits (mostly neuroticism) have been implicated	Most studies were cross‐sectional

* ALE, activation likelihood estimation; GABA, γ‐aminobutyric acid; HPA, hypothalamic pituitary axis; MRI, magnetic resonance imaging; VBM, voxel‐based morphometry.

#### Neurobiologic

The emergence of neuroimaging, particularly magnetic resonance imaging (MRI), has provided insights into brain structural/functional changes (termed “neuroplasticity”) occurring in chronic pain.^60^ Four reviews synthesized literature on brain regions/morphologic brain changes occurring in association with pain and depression. First, Malfliet et al evaluated the relationship between maladaptive cognitive and emotional factors related to pain (including depression) and brain alterations (measured by MRI/functional MRI [fMRI]) in patients with chronic pain. A total of 14 included studies considered depression (measured by self‐reported questionnaires), observing evidence for the following MRI changes: (1) an inverse relationship with resting state functional connectivity in brain areas involved in pain processing (one case‐control study in patients with fibromyalgia, considered “weak” methodologic quality), (2) a positive correlation with parts of the default mode network involved in affective‐cognitive processing (one case‐control study in patients with localized provoked vulvodynia, considered “strong” methodologic quality), and (3) a positive association with pain‐induced activation of the left prefrontal cortex (one case‐control study in patients with irritable bowel syndrome, considered “weak” methodologic quality). However, many studies reported no associations between depression and brain changes, variation in MRI techniques across studies limited the interpretation of the findings, and it is unknown if these changes also occur in people without chronic pain.^54^

Second, Zheng et al used activation likelihood estimation meta‐analysis to identify neural correlations with comorbid pain and depression. Activation likelihood estimation meta‐analysis represents a quantitative approach for pooling neuroimaging metadata.^61^ Twenty‐six studies using MRI, fMRI, or positron emission tomography were included (most considered “poor”/“fair” quality). Meta‐analysis suggested that, in people with pain as the primary diagnosis, concomitant depression associated with increased activation of the right parahippocampal gyrus/amygdala, whereas in people with depression as the primary diagnosis, concomitant pain associated with increased activation of the left superior frontal gyrus and left thalamus. However, only studies reporting statistically significant results were included, with many studies reporting no significant associations.^52^

Third, as amygdala size has been reported to increase in people with major depression, Chen et al synthesized literature on the relationship between emotional stress and MRI‐measured amygdala size in patients with chronic pain versus healthy controls. Emotional stress was captured using outcomes considering both depression and anxiety. Thirteen studies were included; seven reported significant amygdala shrinkage in patients with chronic pain and emotional stress versus controls (Hedges g = −0.73; *P* = 0.02). However, it cannot be established whether these changes are related to depression, anxiety, chronic pain or confounding variables.^51^

Fourth, as gray matter volume alterations are considered key neuroplastic changes in people with chronic pain and depressive symptoms, Ma et al synthesized data from relevant voxel‐based morphometry studies (a method to analyze brain microstructural changes) comparing gray matter changes in patients with chronic pain and associated depression with healthy controls. Eighteen studies were included. Compared with healthy controls, people with chronic pain and depression had increased gray matter volume in the left hippocampus (*P* < 0.005) and decreased gray matter volume in the medial part of the left superior frontal gyrus (*P* < 0.005).^53^

One systematic review explored responses to painful stimuli according to the presence of depression, with Thompson et al conducting a meta‐analysis to compare responses to experimentally induced pain in depressed and healthy groups.^55^ A total of 32 studies were included. Outcomes considered were pain threshold (when pain is first perceived), tolerance (when pain can no longer be tolerated), and intensity. Pain tolerance for high‐intensity noxious stimulation was similar between groups (Hedges g = 0.09, *P* = 0.71; 10 studies), but for low‐intensity stimulation a small, statistically significant higher mean sensory threshold (g = 0.35, *P* = 0.01; nine studies) and pain threshold (g = 0.32, *P* = 0.02; 25 studies) was observed in depressed versus control groups. Although this finding suggests that people with depression may have a higher pain threshold for low‐intensity noxious stimuli, the effect size was small and marked heterogeneity was observed. A proposed conceptual model for this paradoxical finding was that, for low‐intensity painful stimuli, greater attention is devoted to nonpainful stimuli (eg, internal thoughts) reducing mild pain by denying it attention resources but having no impact for higher‐intensity painful stimuli demanding greater attention.

#### Psychosocial

One review considered psychosocial pathways, with Karimi et al examining the extent to which sleep disturbance mediates the relationship between depression and chronic pain. A total of 49 studies were included, with most (37 studies) considered to be of high quality. They reported, with moderate certainty in the evidence, that 12.5% of the total effect of depression on pain could be accounted for by the indirect effect of disturbed sleep, although causality could not be inferred.^56^

#### Multiple mechanisms

Three reviews considered both biologic and psychosocial pathways. First, Antoniou et al synthesized neuroimaging study findings to examine the impact of adverse childhood experiences on neural correlations with chronic pain and depression (either alone or in combination). From 49 included studies, 43 considered adverse childhood experience and major depressive disorder and 4 considered adverse childhood experience and chronic pain (no studies examined adverse childhood experience and major depressive disorder and chronic pain in combination). Most (40 studies) were considered at low risk of bias. Task‐based fMRI results indicated that (compared with people without adverse childhood experiences) those with depression and adverse childhood experiences had functional and structural brain changes in the superior frontal, lingual gyrus, hippocampus, insula, putamen, superior temporal, inferior temporal gyrus, and anterior cerebellum, and those with depression or chronic pain and adverse childhood experiences had changes in the cingulate gyrus, inferior parietal lobule, and precuneus. Although this suggests adverse childhood experiences can lead to brain alterations, without examining changes in people with both chronic pain and depression, their relevance to this relationship is unknown.^57^

Second, Khan et al narratively synthesized findings from twin studies exploring the covariation between pain, depression, and/or anxiety. From 23 included studies, six examined the covariation between pain and depression. Marked heterogeneity was seen in study methods (eg, depression definitions), and all but one were cross‐sectional. Results varied, with one study reporting that the association between pain and depression was “entirely explained” by shared genetic influences, three reporting significant nonshared environmental effects underlying the association, one reporting the association was explained by unmeasured genetic and shared environmental effects, and one reporting no evidence of unmeasured familial factors.^58^ Consequently, the extent to which genetic and environmental factors underlie the association is unclear.

Third, Dresler et al explored the association between migraine and psychiatric disorders (including depression) and its potential mechanisms. For major depression, they reported that studies indicated a bidirectional relationship, with potential mechanisms including shared genes, neurotransmitter changes, shared hypothalamic pituitary adrenal axis involvement, cytokine imbalances, and neuroplasticity.^59^

### Summary

Evidence for causal pathways for the association between pain and depression in IA is limited, with only one longitudinal study showing evidence for a potential causal pathway (suggesting passive pain‐coping behaviors increase the risk of depression). In non‐IA populations, many studies have examined the potential role of neuroplasticity, with various structural/functional brain changes observed in people with pain and depression; however, whether these changes are clinically relevant remains unknown. In non‐IA populations, the clearest evidence relates to the role of sleep disturbance, which is estimated to account for 12.5% of the effect of depression on pain.

## Clinical assessment of pain and depression in IA

### Overview

*This section details evidence on the potential ways to assess pain and depression in adults with IA in routine care. It summarises recommendations from clinical guidelines and systematic review evidence on outcome measures*.

### Guidelines on pain assessments

Most IA guidelines focus on disease activity, providing little guidance for pain assessment.^62^, ^63^, ^64^, ^65^, ^66^ Only one guideline (from EULAR) has specifically focused on IA pain. This emphasizes the need for biopsychosocial pain assessments, considering (1) patients’ views on their pain's cause; (2) pain severity (using a VAS/NRS) and characteristics; (3) current/previous pain treatments; (4) current inflammation/joint damage as pain sources; and (5) pain‐related biologic, psychologic, and social factors.^67^ Undertaking these in a brief outpatient visit/primary care consultation is challenging. A UK guideline, which will cover pain assessments in patients with IA, is currently being produced by the British Society for Rheumatology.^68^

### Guidelines on depression assessments

Although no specific guideline exists for mental health assessments in IA, the EULAR and National Institute For Health and Care Excellence (NICE) IA guidelines highlight the importance of assessing mental health.^63^, ^69^ The NICE RA guideline directs people to NICE “Depression in adults with a chronic physical health problem” guidance, which proposes using a simplified Patient Health Questionnaire (PHQ) 2 version for case identification, asking people who may have depression two questions: (1) “during the last month, have you often been bothered by feeling down, depressed, or hopeless?” and (2) “during the last month, have you often been bothered by having little interest or pleasure in doing things?“^70^ If people answer “yes” to either, it recommends further exploration of symptoms by a practitioner competent in assessing mental health. Within the United States, the Preventive Services Task Force produced a recommendation statement in 2023 on screening for depression in adults. This recommends screening adults for depression in primary care. Although no single approach is advocated, the statement highlights the availability of commonly used screening instruments, including the PHQ, CES‐D, and (in older adults) the Geriatric Depression Scale. It acknowledges the lack of evidence for optimal timing for screening, with a pragmatic approach suggested of screening adults that have not been screened before and using clinical judgment to determine if additional screening of patients at increased risk is warranted.^71^

### Tools to assess pain and depression in IA

We identified 19 systematic reviews evaluating pain and/or depression outcome measures in IA (from 867 references). We have described findings from five key systematic reviews of most clinical relevance (with many considering PROMs in research settings). Full details of all reviews are provided in Table 3. First, Englbrecht et al reviewed literature on measuring pain in IA to generate clinical practice recommendations. Across 51 studies, single pain‐related items, such as the VAS or NRS, were considered well validated and suitable for routine clinical use owing to strong clinimetric properties (convergent validity, responsiveness, and feasibility).^73^ Second, Barnabe et al evaluated validation studies for PROMs in populations at risk of inequities; three studies evaluated the pain VAS, which appeared reliable and feasible across languages (Spanish and Japanese).^72^

**Table 3:** Systematic reviews examining outcome measures for pain and depression in IA

Study, year	Aim	Key findings	Limitation(s)
Pain outcome measures			
Rutter‐Locher et al,^45^ 2023	Evaluate self‐reported pain sensitivity and neuropathic‐like pain in IA	Sixty‐three studies included. The self‐reported questionnaires used were the CSI (11 studies), DN4 (17 studies), LANSS (seven studies), painDETECT (33 studies), and McGill (SF‐McGill [three studies] and McGill [one study]). Pain sensitivity and neuropathic‐like pain in IA reported as having prevalence of 31% to 42%, questionnaire dependent	Significant heterogeneity present
Barnabe et al,^72^ 2022	Identify and rate evidence in validation studies for PROMs in populations at risk of inequity	Considered pain VAS (100 mm or 5‐point scale) and PROMIS pain measures. Three studies considered pain VAS (all 100 mm) and appeared reliable and feasible across languages with strong construct validity linking pain to markers of inflammation (weak correlations with other disease metrics). One study evaluated PROMIS pain measure and showed cross‐cultural adaptability, feasibility, and validity when correlated with established measures	Small number of studies with limited country representation
Ortega‐Avila et al,^75^ 2019	Identify self‐reported outcome measures specific to the foot and ankle in RA and investigate their psychometric properties	Fourteen studies included. Four pain measures identified: Ankle Osteoarthritis Scale, Manchester Foot Pain Disability Index, Rowan Foot Pain Assessment Questionnaire, and Self‐Reported Foot and Ankle Score. Self‐Reported Foot and Ankle Score had the best overall psychometric properties	Incomplete data from some studies
Englbrecht et al,^73^ 2012	Review literature on measuring pain (and its treatment) in IA to generate clinical practice recommendations	Fifty‐one articles included. Data showed that single pain‐related items such as the VAS, NRS, or verbal rating scale provide sufficient clinimetric information and can be recommended as an overall pain measure in clinical practice. Similar results obtained for pain subscales of the AIMS/AIMS2 and bodily pain subscale of SF‐36	Search term limited to “pain measures”; may have overlooked some pain outcome measures
van der Leeden et al,^76^ 2008	Create an instrument inventory of measures for foot function, pain, and related disability in RA (and clinimetric qualities)	A total of 194 included papers. Six feet pain instruments identified including one‐item questions (eg, pain VAS) and a questionnaire (BPI). Nineteen studies and 16 instruments identified for clinimetric evaluation. Six measured both foot pain and disability (two scoring systems; four questionnaires) with varying clinimetric properties and most demonstrating gaps in validation	Search specific to current instruments; may have overlooked older outcome measures
Depression outcome measures			
Victoria et al,^74^ 2023	Determine most accurate depression scale in RA	Twenty‐eight studies included. Eleven tools evaluated, of which the CES‐D (and its variations) were used most frequently and considered to have the best psychometric properties, including internal consistency, sensitivity, and specificity	Methodologic heterogeneity across studies
Dickens et al,^77^ 2002	Examine the association between RA and depression (including the influence of the depression assessment method)	Twelve studies included. Ten used a single measure of depression; three used HADS (other measures include GCS, PDI, IDD, POMS, CES‐D, AIMS, and Kasl D). The effect sizes obtained from studies using HADS were different from those using other methods, with studies using HADS finding patients with RA to be more depressed than studies using other measures	Historical review
Both pain and depression outcome measures			
Teuwen et al,^78^ 2023	Describe the use of PROMIS measures in clinical studies involving people with RA or axial SpA	Twenty‐nine studies included. PROMIS Pain Interference (17 studies) consistently indicated poor health outcomes and was employed in various formats (eg, item banks, computer adaptive tests, and short forms) highlighting its adaptability across study designs. PROMIS Depression (12 studies) similarly was applied in various forms. A greater standardization in the use of PROMIS measures in clinical studies is required	Psychometric properties of PROMIS measures were not reviewed
Hansen et al,^79^ 2022	Identify the use of outcome domains and outcome measures across all self‐management trials in IA	Thirty‐eight studies included. Twenty‐five trials considered pain; pain measures included VAS (16), NRS (6), AIMS2 (2), and HAQ (1). A total of 64% of trials measuring pain used VAS. Sixteen trials considered depression/anxiety. Depression measures included HADS (9), CES‐D (6), AIMS (2), DS (1), and the Zung depression scale (1). A total of 47% of trials measuring depression/anxiety used HADS	Excluded nonvalidated measures
Küçükdeveci et al,^80^ 2021	Describe RA PROMs used over the past 20 years and their performance metrics	A total of 496 articles were included and 125 PROMs identified. Twenty pain PROMs identified: 4 had moderate psychometric evidence (AIMS, AIMS‐2, Nottingham Health Profile, and SF‐36), 10 weak, and 6 absent. Four PROMs were depression specific; only one had moderate psychometric evidence (HADS depression subscale)	Commonly used outcome measures (ie, pain VAS and PHQ‐9) not included
Minnock et al,^81^ 2018	Identify patient outcomes measured in RA studies that reported nursing interventions	Seven RCTs and three observational studies included. Fifty‐nine measurement instruments were identified, including four pain measures (5‐point scale, VAS, AIMS, and RAID). Four depression scales identified (AIMS, NHP, SF‐12 MCS, and HADS)	Qualitative studies were excluded
Tang et al,^82^ 2017	Review literature on RA PROM data and identify measures implemented in Japan	A total of 100 articles included. Of 23 studies measuring pain, 21 used the VAS. Other validated PROMs were not identified for pain. Eight PROMs were used for depression. The Zung SDS was the most frequently reported, followed by the BDI‐2 and HAM‐D	Search only conducted in two databases (PubMed and Ichushi Web)
Kilic et al,^83^ 2016	Assess frequency of PROM use in RA studies (2013–2015) and compare results with a previous systematic review (2005–2007)	A total of 250 articles included. Pain was reported in 40.0% of studies, most of these used VAS/NRS (89.0%). Pain reporting reduced from previous review (previously 55.9%). Psychologic status reported in 9.6% of studies. HADS (25%) and BDI (25%) most used. There was an increase in the use of depression measures from previous review (previously reported at 7.3%)	Only used one database (PubMed) spanning 2 years

*AIMS, Arthritis Impact Measurement Scale; BDI, Becks Depression Inventory; BPI, Bodily Pain Index; CES‐D, Center for Epidemiological Studies Depression; CSI, Central Sensitization Inventory; DN4, Douleur Neuropathique 4; DS, Depression Scale; GCS, Glasgow Depression Scale; HADS‐D, Hospital Anxiety Depression Scale‐Depression subscale; HAM‐D, Hamilton Depression Rating Scale; HAQ, Health Assessment Questionnaire; IA, inflammatory arthritis; IDD, Inventory to Diagnose Depression; LANSS, Leeds Assessment of Neuropathic Symptoms and Signs; NHP, Nottingham Health Profile; NRS, Numeric Rating Scale; PDI, Perinatal Depression Inventory; PHQ‐9, Patient Health Questionnaire‐9; POMS, Profile of Mood States; PROM, patient‐reported outcome measure; PROMIS, Patient‐Reported Outcomes Measurement Information System; RA, rheumatoid arthritis; RAID, Rapid Assessment, Interface, and Discharge; SDS, Self‐Rating Depression Scale; SF, Short Form; SF‐12 MCS, Short Form 12 Mental Component Score; SF‐36, Short Form 36; SpA, spondyloarthropathy; VAS, Visual Analog Scale.

Third, Rutter‐Locher et al evaluated the prevalence of pain sensitivity/neuropathic‐like pain in studies using PROMs. A total of 11 studies used the Central Sensitisation Inventory, 17 studies used the Douleur Neuropathique 4 questionnaire, 7 studies used the Leeds Assessment of Neuropathic Symptoms and Signs questionnaire, 33 studies used the painDETECT questionnaire, and 4 studies used the McGill questionnaire. The prevalence of pain sensitivity and neuropathic‐like pain ranged from 31% to 42%, highlighting the need to consider the type of pain in assessments.^45^

Fourth, Victoria et al sought to determine the most accurate depression scale in patients with RA. Across the 28 studies, 11 tools were evaluated, of which the CES‐D (and its variations) were used most frequently and considered to have the best psychometric properties.^74^ Finally, Küçükdeveci et al synthesized evidence of performance (eg, validity, ability to detect meaningful differences, and feasibility) of PROMs in RA to help clinicians make informed choices about which to use for a given purpose. A process to assess the quality of reported psychometric evidence for PROMs (based on the Outcome Measures in RA Clinical Trials filter) was applied. Twenty PROMs to assess pain were identified: four (AIMS, AIMS‐2, Nottingham Health Profile, and SF‐36) were considered to have moderate, 10 weak, and six absent underpinning psychometric evidence. Of 23 PROMs to assess emotional function/mental health, four were depression specific, and only one was considered to have moderate psychometric evidence (HADS depression subscale). Notably, the widely used pain VAS/NRS and PHQ‐9 were not included.^80^

### Summary

Current IA guidelines lack detailed recommendations about the assessment of pain and depression and, despite a range of PROMs existing to assess these in patients with IA, the evidence for their psychometric properties is often considered limited. However, it is our perspective that, in view of the dominance of pain in patients with IA, all IA consultations should incorporate the use of PROMs to routinely measure pain (eg, an NRS, which has robust supportive evidence and is quick to complete) and screening for depression where relevant. In our own clinical practice, the use of electronic PROMs to achieve this is highly acceptable to patients.^84^

## Future research

Our review has highlighted two research needs. First is a requirement to better understand the causal pathways underpinning the bidirectional relationship between pain and depression in IA. These are likely to include biologic (eg, neuroplasticity), psychologic (eg, pain‐coping strategies), and social (eg, work impact) factors. Understanding pain mechanisms is essential to develop novel pain therapies. However, regardless of causality and mechanisms, there is a compelling argument to routinely assess and manage pain and depression in patients with IA, as improving both is likely to have beneficial effects on patients’ lives. Therefore, the second research need is to explore how to best integrate pain assessments and depression screening into routine IA care in a manner that is acceptable to patients and clinicians and fair. Although digital systems show promise in achieving this, they have the potential to worsen care inequalities; for instance, older adults (in whom IA is more common^85^) are less likely to have internet access^86^ or the skills necessary to navigate online resources independently.^87^ Ensuring that groups of people are not left behind in digitally evolving health care systems is crucial.

## Conclusions

Many longitudinal studies demonstrate a relationship between pain and depression in people with IA, which appears to be bidirectional. Although the causal pathways underpinning this are unclear, and the effect sizes are small to modest, the prevalent nature of chronic pain and depression in patients with IA supports the role of routinely assessing both issues in outpatient consultations and implementing systemic care approaches to effectively manage them. Further research is needed to understand how to best achieve this in a manner that is feasible and acceptable to patients and clinicians.

## AUTHOR CONTRIBUTIONS

All authors were involved in drafting the article or revising it critically for important intellectual content, and all authors approved the final version to be published.

REFERENCES1

Radawski
C
, 
Genovese
MC
, 
Hauber
B
, et al. Patient perceptions of unmet medical need in rheumatoid arthritis: a cross‐sectional survey in the USA. Rheumatol Ther
2019;6(3):461–471.31385264
10.1007/s40744-019-00168-5PMC67026172

Taylor
P
, 
Manger
B
, 
Alvaro‐Gracia
J
, et al. Patient perceptions concerning pain management in the treatment of rheumatoid arthritis. J Int Med Res
2010;38(4):1213–1224.20925993
10.1177/1473230010038004023

Heiberg
T
, 
Kvien
TK
. Preferences for improved health examined in 1,024 patients with rheumatoid arthritis: pain has highest priority. Arthritis Rheum
2002;47(4):391–397.12209485
10.1002/art.105154

McWilliams
DF
, 
Dawson
O
, 
Young
A
, et al. Discrete trajectories of resolving and persistent pain in people with rheumatoid arthritis despite undergoing treatment for inflammation: results from three UK cohorts. J Pain
2019;20(6):716–727.30658176
10.1016/j.jpain.2019.01.0015

Pisaniello
HL
, 
Lester
S
, 
Russell
O
, et al. Trajectories of self‐reported pain‐related health outcomes and longitudinal effects on medication use in rheumatoid arthritis: a prospective cohort analysis using the Australian Rheumatology Association Database (ARAD). RMD Open
2023;9(3):e002962.37507204
10.1136/rmdopen-2022-002962PMC103916336

Strand
V
, 
Deodhar
A
, 
Alten
R
, et al. Pain and fatigue in patients with ankylosing spondylitis treated with tumor necrosis factor inhibitors: multinational real‐world findings. J Clin Rheumatol
2021;27(8):e446–e455.32826654
10.1097/RHU.0000000000001544PMC86128857

Conaghan
PG
, 
Alten
R
, 
Deodhar
A
, et al. Relationship of pain and fatigue with health‐related quality of life and work in patients with psoriatic arthritis on TNFi: results of a multi‐national real‐world study. RMD Open
2020;6(2):e001240.32611650
10.1136/rmdopen-2020-001240PMC74251928

Hirano
F
, 
van der Heijde
D
, 
van Gaalen
FA
, et al. Determinants of the patient global assessment of well‐being in early axial spondyloarthritis: 5‐year longitudinal data from the DESIR cohort. Rheumatology (Oxford)
2021;60(1):316–321.32766697
10.1093/rheumatology/keaa353PMC77853129

Euesden
J
, 
Matcham
F
, 
Hotopf
M
, et al. The relationship between mental health, disease severity, and genetic risk for depression in early rheumatoid arthritis. Psychosom Med
2017;79(6):638–645.28282363
10.1097/PSY.0000000000000462PMC563842110

Pollard
LC
, 
Choy
EH
, 
Gonzalez
J
, et al. Fatigue in rheumatoid arthritis reflects pain, not disease activity. Rheumatology (Oxford)
2006;45(7):885–889.16449363
10.1093/rheumatology/kel02111

Scott
IC
, 
Daud
N
, 
Bailey
J
, et al. Gabapentinoid use and the risk of fractures in patients with inflammatory arthritis: nested case‐control study in the Clinical Practice Research Datalink Aurum. BMC Med
2024;22(1):575.39663522
10.1186/s12916-024-03774-5PMC1163603112

Cox
N
, 
Mallen
CD
, 
Scott
IC
. Pharmacological pain management in patients with rheumatoid arthritis: a narrative literature review. BMC Med
2025;23(1):54.39881356
10.1186/s12916-025-03870-0PMC1178077913
Arthritis Research UK
. A research roadmap for pain. Accessed November 1, 2025. https://www.versusarthritis.org/media/1672/research-roadmap-pain.pdf
14

Matcham
F
, 
Rayner
L
, 
Steer
S
, et al. The prevalence of depression in rheumatoid arthritis: a systematic review and meta‐analysis: reply. Rheumatology (Oxford)
2014;53(3):578–579.10.1093/rheumatology/ket4392440257915

Vestergaard
SB
, 
Esbensen
BA
, 
Klausen
JM
, et al. Prevalence of anxiety and depression and the association with self‐management behaviour in >12 000 patients with inflammatory rheumatic disease: a cross‐sectional nationwide study. RMD Open
2024;10(1):e003412.38253596
10.1136/rmdopen-2023-003412PMC1080650016
World Health Organization
. Depressive disorder (depression). Published March 31, 2023. Accessed July 1, 2025. https://www.who.int/news-room/fact-sheets/detail/depression
17

Mattey
DL
, 
Dawes
PT
, 
Hassell
AB
, et al. Effect of psychological distress on continuation of anti‐tumor necrosis factor therapy in patients with rheumatoid arthritis. J Rheumatol
2010;37(10):2021–2024.20682674
10.3899/jrheum.10005018

Ho
RCM
, 
Fu
EHY
, 
Chua
ANC
, et al. Clinical and psychosocial factors associated with depression and anxiety in Singaporean patients with rheumatoid arthritis. Int J Rheum Dis
2011;14(1):37–47.21303480
10.1111/j.1756-185X.2010.01591.x19

Ang
DC
, 
Choi
H
, 
Kroenke
K
, et al. Comorbid depression is an independent risk factor for mortality in patients with rheumatoid arthritis. J Rheumatol
2005;32(6):1013–1019.15940760
20

Kojima
M
, 
Kojima
T
, 
Suzuki
S
, et al. Alexithymia, depression, inflammation, and pain in patients with rheumatoid arthritis. Arthritis Care Res (Hoboken)
2014;66(5):679–686.24127403
10.1002/acr.2220321

Xu
X
, 
Shen
B
, 
Zhang
A
, et al. Anxiety and depression correlate with disease and quality‐of‐life parameters in Chinese patients with ankylosing spondylitis. Patient Prefer Adherence
2016;10:879–885.27284241
10.2147/PPA.S86612PMC488192822

Ben Tekaya
A
, 
Mahmoud
I
, 
Hamdı
I
, et al. Depression and anxiety in spondyloarthritis: prevalence and relationship with clinical parameters and self‐reported outcome measures. Turk Psikiyatri Derg
2019;30(2):90–98.31487374
23

Adnine
A
, 
Nadiri
K
, 
Soussan
I
, et al. Mental health problems experienced by patients with rheumatic diseases during COVID‐19 pandemic. Curr Rheumatol Rev
2021;17(3):303–311.33504309
10.2174/157339711766621012712454424

Swinscow
TDV
. Correlation and regression. In: Statistics at Square One. 9th ed.
BMJ Publishing Group; 1997.25

LaMorte
WW
. PH717 Module 9: correlation and regression evaluating association between two continuous variables. Accessed November 1, 2025. https://www.bu.edu/sph/online-mph-and-teaching-public-health/
26

Gialouri
CG
, 
Evangelatos
G
, 
Zhao
SS
, et al. Depression and anxiety in a real‐world psoriatic arthritis longitudinal study: should we focus more on patients’ perception?
Clin Exp Rheumatol
2023;41(1):159–165.35819812
10.55563/clinexprheumatol/8qxo8027

Chaney
JM
, 
Mullins
LL
, 
Wagner
JL
, et al. A longitudinal examination of causal attributions and depression symptomatology in rheumatoid arthritis. Rehabil Psychol
2004;49(2):126–133.28

Graham‐Engeland
JE
, 
Zawadzki
MJ
, 
Slavish
DC
, et al. Depressive symptoms and momentary mood predict momentary pain among rheumatoid arthritis patients. Ann Behav Med
2016;50(1):12–23.26272466
10.1007/s12160-015-9723-2PMC474413029

Ward
MM
. Are patient self‐report measures of arthritis activity confounded by mood? A longitudinal study of patients with rheumatoid arthritis. J Rheumatol
1994;21(6):1046–1050.7932413
30

Snoeck Henkemans
SVJ
, 
Vis
M
, 
Koc
GH
, et al. Association between depression and anxiety and inability to achieve remission in rheumatoid arthritis and psoriatic arthritis. Rheumatology (Oxford) Published online November 6, 2024. 10.1093/rheumatology/keae621
3967279031

Rathbun
AM
, 
England
BR
, 
Mikuls
TR
, et al. Relationship between depression and disease activity in United States veterans with early rheumatoid arthritis receiving methotrexate. J Rheumatol
2021;48(6):813–820.33191277
10.3899/jrheum.200743PMC812189832

Wolfe
F
, 
Hawley
DJ
. The relationship between clinical activity and depression in rheumatoid arthritis. J Rheumatol
1993;20(12):2032–2037.8014930
33

McQuillan
J
, 
Andersen
JA
, 
Berdahl
TA
, et al. Associations of rheumatoid arthritis and depressive symptoms over time: are there differences by education, race/ethnicity, and gender?
Arthritis Care Res (Hoboken)
2022;74(12):2050–2058.34121353
10.1002/acr.2473034

Conner
TS
, 
Tennen
H
, 
Zautra
AJ
, et al. Coping with rheumatoid arthritis pain in daily life: within‐person analyses reveal hidden vulnerability for the formerly depressed. Pain
2006;126(1‐3):198–209.16904829
10.1016/j.pain.2006.06.03335

Husted
JA
, 
Tom
BD
, 
Farewell
VT
, et al. Longitudinal study of the bidirectional association between pain and depressive symptoms in patients with psoriatic arthritis. Arthritis Care Res (Hoboken)
2012;64(5):758–765.22231988
10.1002/acr.2160236

Brown
GK
. A causal analysis of chronic pain and depression. J Abnorm Psychol
1990;99(2):127–137.2348006
10.1037//0021-843x.99.2.12737

Smedstad
LM
, 
Vaglum
P
, 
Moum
T
, et al. The relationship between psychological distress and traditional clinical variables: a 2 year prospective study of 216 patients with early rheumatoid arthritis. Br J Rheumatol
1997;36(12):1304–1311.9448592
10.1093/rheumatology/36.12.130438

Odegård
S
, 
Finset
A
, 
Mowinckel
P
, et al. Pain and psychological health status over a 10‐year period in patients with recent onset rheumatoid arthritis. Ann Rheum Dis
2007;66(9):1195–1201.17392351
10.1136/ard.2006.064287PMC195516139

Hawley
DJ
, 
Wolfe
F
. Anxiety and depression in patients with rheumatoid arthritis: a prospective study of 400 patients. J Rheumatol
1988;15(6):932–941.3418643
40
International Association for the Study of Pain
. IASP Announces Revised Definition of Pain. Published July 16, 2020. Accessed July 1, 2025. https://www.iasp-pain.org/publications/iasp-news/iasp-announces-revised-definition-of-pain/
41

Raja
SN
, 
Carr
DB
, 
Cohen
M
, et al. The revised International Association for the Study of Pain definition of pain: concepts, challenges, and compromises. Pain
2020;161(9):1976–1982.32694387
10.1097/j.pain.0000000000001939PMC768071642
International Association for the Study of Pain
. Terminology. Accessed August 1, 2024. https://www.iasp-pain.org/resources/terminology/?ItemNumber=1698#Pain
43

Al Mohamad
F
, 
Rios Rodriguez
V
, 
Haibel
H
, et al. Association of nociplastic and neuropathic pain components with the presence of residual symptoms in patients with axial spondyloarthritis receiving biological disease‐modifying antirheumatic drugs. RMD Open
2024;10(1):e004009.38360039
10.1136/rmdopen-2023-004009PMC1087553444

Khot
S
, 
Tackley
G
, 
Choy
E
. How to distinguish non‐inflammatory from inflammatory pain in RA?
Curr Rheumatol Rep
2024;26(12):403–413.39120749
10.1007/s11926-024-01159-4PMC1152791145

Rutter‐Locher
Z
, 
Arumalla
N
, 
Norton
S
, et al. A systematic review and meta‐analysis of questionnaires to screen for pain sensitisation and neuropathic like pain in inflammatory arthritis. Semin Arthritis Rheum
2023;61:152207.37163841
10.1016/j.semarthrit.2023.15220746

Duffield
SJ
, 
Miller
N
, 
Zhao
S
, et al. Concomitant fibromyalgia complicating chronic inflammatory arthritis: a systematic review and meta‐analysis. Rheumatology (Oxford)
2018;57(8):1453–1460.29788461
10.1093/rheumatology/key112PMC605565147

Freynhagen
R
, 
Parada
HA
, 
Calderon‐Ospina
CA
, et al. Current understanding of the mixed pain concept: a brief narrative review. Curr Med Res Opin
2019;35(6):1011–1018.30479161
10.1080/03007995.2018.155204248

Jesulola
E
, 
Micalos
P
, 
Baguley
IJ
. Understanding the pathophysiology of depression: from monoamines to the neurogenesis hypothesis model ‐ are we there yet?
Behav Brain Res
2018;341:79–90.29284108
10.1016/j.bbr.2017.12.02549

Ionescu
CE
, 
Popescu
CC
, 
Agache
M
, et al. Depression in rheumatoid arthritis: a narrative review‐diagnostic challenges, pathogenic mechanisms and effects. Medicina (Kaunas)
2022;58(11):1637.36422176
10.3390/medicina58111637PMC969666150

Brown
GK
, 
Nicassio
PM
, 
Wallston
KA
. Pain coping strategies and depression in rheumatoid arthritis. J Consult Clin Psychol
1989;57(5):652–657.2794186
10.1037//0022-006x.57.5.65251

Chen
MH
, 
Sun
CK
, 
Lin
IM
, et al. Size reduction of the right amygdala in chronic pain patients with emotional stress: a systematic review and meta‐analysis. Pain Med
2023;24(5):556–565.36308460
10.1093/pm/pnac16252

Zheng
CJ
, 
Van Drunen
S
, 
Egorova‐Brumley
N
. Neural correlates of co‐occurring pain and depression: an activation‐likelihood estimation (ALE) meta‐analysis and systematic review. Transl Psychiatry
2022;12(1):196.35545623
10.1038/s41398-022-01949-3PMC909571953

Ma
T
, 
Ji
YY
, 
Yan
LF
, et al. Gray matter volume abnormality in chronic pain patients with depressive symptoms: a systemic review and meta‐analysis of voxel‐based morphometry studies. Front Neurosci
2022;16:826759.35733934
10.3389/fnins.2022.826759PMC920740954

Malfliet
A
, 
Coppieters
I
, 
Van Wilgen
P
, et al. Brain changes associated with cognitive and emotional factors in chronic pain: a systematic review. Eur J Pain
2017;21(5):769–786.28146315
10.1002/ejp.100355

Thompson
T
, 
Correll
CU
, 
Gallop
K
, et al. Is pain perception altered in people with depression? A systematic review and meta‐analysis of experimental pain research. J Pain
2016;17(12):1257–1272.27589910
10.1016/j.jpain.2016.08.00756

Karimi
R
, 
Mallah
N
, 
Scherer
R
, et al. Sleep quality as a mediator of the relation between depression and chronic pain: a systematic review and meta‐analysis. Br J Anaesth
2023;130(6):747–762.37059623
10.1016/j.bja.2023.02.03657

Antoniou
G
, 
Lambourg
E
, 
Steele
JD
, et al. The effect of adverse childhood experiences on chronic pain and major depression in adulthood: a systematic review and meta‐analysis. Br J Anaesth
2023;130(6):729–746.37087334
10.1016/j.bja.2023.03.008PMC1025113058

Khan
WU
, 
Michelini
G
, 
Battaglia
M
. Twin studies of the covariation of pain with depression and anxiety: a systematic review and re‐evaluation of critical needs. Neurosci Biobehav Rev
2020;111:135–148.31954722
10.1016/j.neubiorev.2020.01.01559

Dresler
T
, 
Caratozzolo
S
, 
Guldolf
K
, et al; European Headache Federation School of Advanced Studies (EHF‐SAS). Understanding the nature of psychiatric comorbidity in migraine: a systematic review focused on interactions and treatment implications. J Headache Pain
2019;20(1):51.31072313
10.1186/s10194-019-0988-xPMC673426160

Martucci
KT
, 
Ng
P
, 
Mackey
S
. Neuroimaging chronic pain: what have we learned and where are we going?
Future Neurol
2014;9(6):615–626.28163658
10.2217/FNL.14.57PMC528982461

Kirby
LAJ
, 
Robinson
JL
. Affective mapping: an activation likelihood estimation (ALE) meta‐analysis. Brain Cogn
2017;118:137–148.26074298
10.1016/j.bandc.2015.04.00662

Fraenkel
L
, 
Bathon
JM
, 
England
BR
, et al. 2021 American College of Rheumatology guideline for the treatment of rheumatoid arthritis. Arthritis Rheumatol
2021;73(7):1108–1123.34101376
10.1002/art.4175263
National Institute for Health and Care Excellence
. Rheumatoid arthritis in adults: management. Updated October 12, 2020. Accessed November 11, 2025. https://www.nice.org.uk/guidance/ng100
64
National Institute for Health and Care Excellence
. Spondyloarthritis in over 16s: diagnosis and management. Updated June 2, 2017. Accessed November 1, 2025. https://www.nice.org.uk/guidance/ng65
2835042865

Singh
JA
, 
Guyatt
G
, 
Ogdie
A
, et al. Special article: 2018 American College of Rheumatology/National Psoriasis Foundation guideline for the treatment of psoriatic arthritis. Arthritis Rheumatol
2019;71(1):5–32.30499246
10.1002/art.40726PMC821833366

Ward
MM
, 
Deodhar
A
, 
Gensler
LS
, et al. 2019 Update of the American College of Rheumatology/Spondylitis Association of America/Spondyloarthritis Research and Treatment Network recommendations for the treatment of ankylosing spondylitis and nonradiographic axial spondyloarthritis. Arthritis Rheumatol
2019;71(10):1599–1613.31436036
10.1002/art.41042PMC676488267

Geenen
R
, 
Overman
CL
, 
Christensen
R
, et al. EULAR recommendations for the health professional's approach to pain management in inflammatory arthritis and osteoarthritis. Ann Rheum Dis
2018;77(6):797–807.29724726
10.1136/annrheumdis-2017-21266268

Scott
IC
, 
Babatunde
O
, 
Barker
C
, et al. Pain management in people with inflammatory arthritis: British Society for Rheumatology guideline scope. Rheumatol Adv Pract
2024;8(4):rkae128.39563967
10.1093/rap/rkae128PMC1157341369

Nikiphorou
E
, 
Santos
EJF
, 
Marques
A
, et al. 2021 EULAR recommendations for the implementation of self‐management strategies in patients with inflammatory arthritis. Ann Rheum Dis
2021;80(10):1278–1285.33962964
10.1136/annrheumdis-2021-220249PMC845809370
National Institute for Health and Care Excellence
. Depression in adults with a chronic physical health problem: recognition and management. Published October 28, 2009. Accessed November 1, 2025. https://www.nice.org.uk/guidance/cg91
3980801571

Barry
MJ
, 
Nicholson
WK
, 
Silverstein
M
, et al; US Preventive Services Task Force. Screening for depression and suicide risk in adults: US Preventive Services Task Force recommendation statement. JAMA
2023;329(23):2057–2067.37338872
10.1001/jama.2023.929772

Barnabe
C
, 
Wattiaux
A
, 
Petkovic
J
, et al. Validation studies of rheumatoid arthritis patient‐reported outcome measures in populations at risk for inequity: a systematic review and analysis using the OMERACT summary of measurement properties equity table. Semin Arthritis Rheum
2022;55:152029.35640489
10.1016/j.semarthrit.2022.15202973

Englbrecht
M
, 
Tarner
IH
, 
van der Heijde
DM
, et al. Measuring pain and efficacy of pain treatment in inflammatory arthritis: a systematic literature review. J Rheumatol Suppl
2012;90:3–10.22942322
10.3899/jrheum.12033574

Victoria
MA
, 
Lucio
VR
, 
Cristina
HD
. Patient self‐reported instruments for assessing symptoms in rheumatoid arthritis. Rheumatol Int
2023;43(10):1781–1790.37322354
10.1007/s00296-023-05355-w75

Ortega‐Avila
AB
, 
Ramos‐Petersen
L
, 
Cervera‐Garvi
P
, et al. Systematic review of the psychometric properties of patient‐reported outcome measures for rheumatoid arthritis in the foot and ankle. Clin Rehabil
2019;33(11):1788–1799.31291785
10.1177/026921551986232876

van der Leeden
M
, 
Steultjens
MPM
, 
Terwee
CB
, et al. A systematic review of instruments measuring foot function, foot pain, and foot‐related disability in patients with rheumatoid arthritis. Arthritis Rheum
2008;59(9):1257–1269.18759256
10.1002/art.2401677

Dickens
C
, 
McGowan
L
, 
Clark‐Carter
D
, et al. Depression in rheumatoid arthritis: a systematic review of the literature with meta‐analysis. Psychosom Med
2002;64(1):52–60.11818586
10.1097/00006842-200201000-0000878

Teuwen
MMH
, 
Knaapen
IRE
, 
Vliet Vlieland
TPM
, et al. The use of PROMIS measures in clinical studies in patients with inflammatory arthritis: a systematic review. Qual Life Res
2023;32(10):2731–2749.37103773
10.1007/s11136-023-03422-0PMC1047417579

Hansen
CW
, 
Esbensen
BA
, 
de Thurah
A
, et al. Outcome measures in rheumatology applied in self‐management interventions targeting people with inflammatory arthritis: a systematic review of outcome domains and measurement instruments. Semin Arthritis Rheum
2022;54:151995.35397237
10.1016/j.semarthrit.2022.15199580

Küçükdeveci
AA
, 
Elhan
AH
, 
Erdoğan
BD
, et al. Use and detailed metric properties of patient‐reported outcome measures for rheumatoid arthritis: a systematic review covering two decades. RMD Open
2021;7(2):e001707.34376556
10.1136/rmdopen-2021-001707PMC835616381

Minnock
P
, 
McKee
G
, 
Kelly
A
, et al. Nursing sensitive outcomes in patients with rheumatoid arthritis: a systematic literature review. Int J Nurs Stud
2018;77:115–129.29080437
10.1016/j.ijnurstu.2017.09.00582

Tang
AC
, 
Kim
H
, 
Crawford
B
, et al. The use of patient reported outcome measures for rheumatoid arthritis in Japan: a systematic literature review. Open Rheumatol J
2017;11(1):43–52.28553419
10.2174/1874312901711010043PMC542769283

Kilic
L
, 
Erden
A
, 
Bingham
CO
III
, et al. The reporting of patient‐reported outcomes in studies of patients with rheumatoid arthritis: a systematic review of 250 articles. J Rheumatol
2016;43(7):1300–1305.27084908
10.3899/jrheum.15117784

Cox
N
, 
Kettle
C
, 
Wang
H
, et al. E041 Feasibility and acceptability of electronic patient reported outcome measures in the routine care of people with inflammatory arthritis: Haywood Arthritis Portal study. Rheumatology
2024;63(Supplement_1):keae163.269.85

Scott
IC
, 
Whittle
R
, 
Bailey
J
, et al. Rheumatoid arthritis, psoriatic arthritis, and axial spondyloarthritis epidemiology in England from 2004 to 2020: an observational study using primary care electronic health record data. Lancet Reg Health Eur
2022;23:100519.36246147
10.1016/j.lanepe.2022.100519PMC955703486
Office for National Statistics
. Internet access ‐ households and individuals QMI. Updated August 8, 2019. Accessed November 1, 2025. https://www.ons.gov.uk/peoplepopulationandcommunity/householdcharacteristics/homeinternetandsocialmediausage/methodologies/internetaccesshouseholdsandindividualsqmi
87
Lloyds Bank
. UK Consumer Digital Index. Accessed November 1, 2025. https://www.lloydsbank.com/banking‐with‐us/whats‐happening/consumer‐digital‐index.html


## Supporting information


**Disclosure form**.

Supplementary Figure 1:

## References

[1] Radawski C , Genovese MC , Hauber B , et al. Patient perceptions of unmet medical need in rheumatoid arthritis: a cross‐sectional survey in the USA. Rheumatol Ther 2019;6(3):461–471. 31385264 10.1007/s40744-019-00168-5 PMC6702617

[2] Taylor P , Manger B , Alvaro‐Gracia J , et al. Patient perceptions concerning pain management in the treatment of rheumatoid arthritis. J Int Med Res 2010;38(4):1213–1224. 20925993 10.1177/147323001003800402

[3] Heiberg T , Kvien TK . Preferences for improved health examined in 1,024 patients with rheumatoid arthritis: pain has highest priority. Arthritis Rheum 2002;47(4):391–397. 12209485 10.1002/art.10515

[4] McWilliams DF , Dawson O , Young A , et al. Discrete trajectories of resolving and persistent pain in people with rheumatoid arthritis despite undergoing treatment for inflammation: results from three UK cohorts. J Pain 2019;20(6):716–727. 30658176 10.1016/j.jpain.2019.01.001

[5] Pisaniello HL , Lester S , Russell O , et al. Trajectories of self‐reported pain‐related health outcomes and longitudinal effects on medication use in rheumatoid arthritis: a prospective cohort analysis using the Australian Rheumatology Association Database (ARAD). RMD Open 2023;9(3):e002962. 37507204 10.1136/rmdopen-2022-002962 PMC10391633

[6] Strand V , Deodhar A , Alten R , et al. Pain and fatigue in patients with ankylosing spondylitis treated with tumor necrosis factor inhibitors: multinational real‐world findings. J Clin Rheumatol 2021;27(8):e446–e455. 32826654 10.1097/RHU.0000000000001544 PMC8612885

[7] Conaghan PG , Alten R , Deodhar A , et al. Relationship of pain and fatigue with health‐related quality of life and work in patients with psoriatic arthritis on TNFi: results of a multi‐national real‐world study. RMD Open 2020;6(2):e001240. 32611650 10.1136/rmdopen-2020-001240 PMC7425192

[8] Hirano F , van der Heijde D , van Gaalen FA , et al. Determinants of the patient global assessment of well‐being in early axial spondyloarthritis: 5‐year longitudinal data from the DESIR cohort. Rheumatology (Oxford) 2021;60(1):316–321. 32766697 10.1093/rheumatology/keaa353 PMC7785312

[9] Euesden J , Matcham F , Hotopf M , et al. The relationship between mental health, disease severity, and genetic risk for depression in early rheumatoid arthritis. Psychosom Med 2017;79(6):638–645. 28282363 10.1097/PSY.0000000000000462 PMC5638421

[10] Pollard LC , Choy EH , Gonzalez J , et al. Fatigue in rheumatoid arthritis reflects pain, not disease activity. Rheumatology (Oxford) 2006;45(7):885–889. 16449363 10.1093/rheumatology/kel021

[11] Scott IC , Daud N , Bailey J , et al. Gabapentinoid use and the risk of fractures in patients with inflammatory arthritis: nested case‐control study in the Clinical Practice Research Datalink Aurum. BMC Med 2024;22(1):575. 39663522 10.1186/s12916-024-03774-5 PMC11636031

[12] Cox N , Mallen CD , Scott IC . Pharmacological pain management in patients with rheumatoid arthritis: a narrative literature review. BMC Med 2025;23(1):54. 39881356 10.1186/s12916-025-03870-0 PMC11780779

[13] Arthritis Research UK . A research roadmap for pain. Accessed November 1, 2025. https://www.versusarthritis.org/media/1672/research-roadmap-pain.pdf

[14] Matcham F , Rayner L , Steer S , et al. The prevalence of depression in rheumatoid arthritis: a systematic review and meta‐analysis: reply. Rheumatology (Oxford) 2014;53(3):578–579. 10.1093/rheumatology/ket439 24402579

[15] Vestergaard SB , Esbensen BA , Klausen JM , et al. Prevalence of anxiety and depression and the association with self‐management behaviour in >12 000 patients with inflammatory rheumatic disease: a cross‐sectional nationwide study. RMD Open 2024;10(1):e003412. 38253596 10.1136/rmdopen-2023-003412 PMC10806500

[16] World Health Organization . Depressive disorder (depression). Published March 31, 2023. Accessed July 1, 2025. https://www.who.int/news-room/fact-sheets/detail/depression

[17] Mattey DL , Dawes PT , Hassell AB , et al. Effect of psychological distress on continuation of anti‐tumor necrosis factor therapy in patients with rheumatoid arthritis. J Rheumatol 2010;37(10):2021–2024. 20682674 10.3899/jrheum.100050

[18] Ho RCM , Fu EHY , Chua ANC , et al. Clinical and psychosocial factors associated with depression and anxiety in Singaporean patients with rheumatoid arthritis. Int J Rheum Dis 2011;14(1):37–47. 21303480 10.1111/j.1756-185X.2010.01591.x

[19] Ang DC , Choi H , Kroenke K , et al. Comorbid depression is an independent risk factor for mortality in patients with rheumatoid arthritis. J Rheumatol 2005;32(6):1013–1019. 15940760

[20] Kojima M , Kojima T , Suzuki S , et al. Alexithymia, depression, inflammation, and pain in patients with rheumatoid arthritis. Arthritis Care Res (Hoboken) 2014;66(5):679–686. 24127403 10.1002/acr.22203

[21] Xu X , Shen B , Zhang A , et al. Anxiety and depression correlate with disease and quality‐of‐life parameters in Chinese patients with ankylosing spondylitis. Patient Prefer Adherence 2016;10:879–885. 27284241 10.2147/PPA.S86612 PMC4881928

[22] Ben Tekaya A , Mahmoud I , Hamdı I , et al. Depression and anxiety in spondyloarthritis: prevalence and relationship with clinical parameters and self‐reported outcome measures. Turk Psikiyatri Derg 2019;30(2):90–98. 31487374

[23] Adnine A , Nadiri K , Soussan I , et al. Mental health problems experienced by patients with rheumatic diseases during COVID‐19 pandemic. Curr Rheumatol Rev 2021;17(3):303–311. 33504309 10.2174/1573397117666210127124544

[24] Swinscow TDV . Correlation and regression. In: Statistics at Square One. 9th ed. BMJ Publishing Group; 1997.

[25] LaMorte WW . PH717 Module 9: correlation and regression evaluating association between two continuous variables. Accessed November 1, 2025. https://www.bu.edu/sph/online-mph-and-teaching-public-health/

[26] Gialouri CG , Evangelatos G , Zhao SS , et al. Depression and anxiety in a real‐world psoriatic arthritis longitudinal study: should we focus more on patients’ perception? Clin Exp Rheumatol 2023;41(1):159–165. 35819812 10.55563/clinexprheumatol/8qxo80

[27] Chaney JM , Mullins LL , Wagner JL , et al. A longitudinal examination of causal attributions and depression symptomatology in rheumatoid arthritis. Rehabil Psychol 2004;49(2):126–133.

[28] Graham‐Engeland JE , Zawadzki MJ , Slavish DC , et al. Depressive symptoms and momentary mood predict momentary pain among rheumatoid arthritis patients. Ann Behav Med 2016;50(1):12–23. 26272466 10.1007/s12160-015-9723-2 PMC4744130

[29] Ward MM . Are patient self‐report measures of arthritis activity confounded by mood? A longitudinal study of patients with rheumatoid arthritis. J Rheumatol 1994;21(6):1046–1050. 7932413

[30] Snoeck Henkemans SVJ , Vis M , Koc GH , et al. Association between depression and anxiety and inability to achieve remission in rheumatoid arthritis and psoriatic arthritis. Rheumatology (Oxford) Published online November 6, 2024. 10.1093/rheumatology/keae621 39672790

[31] Rathbun AM , England BR , Mikuls TR , et al. Relationship between depression and disease activity in United States veterans with early rheumatoid arthritis receiving methotrexate. J Rheumatol 2021;48(6):813–820. 33191277 10.3899/jrheum.200743 PMC8121898

[32] Wolfe F , Hawley DJ . The relationship between clinical activity and depression in rheumatoid arthritis. J Rheumatol 1993;20(12):2032–2037. 8014930

[33] McQuillan J , Andersen JA , Berdahl TA , et al. Associations of rheumatoid arthritis and depressive symptoms over time: are there differences by education, race/ethnicity, and gender? Arthritis Care Res (Hoboken) 2022;74(12):2050–2058. 34121353 10.1002/acr.24730

[34] Conner TS , Tennen H , Zautra AJ , et al. Coping with rheumatoid arthritis pain in daily life: within‐person analyses reveal hidden vulnerability for the formerly depressed. Pain 2006;126(1‐3):198–209. 16904829 10.1016/j.pain.2006.06.033

[35] Husted JA , Tom BD , Farewell VT , et al. Longitudinal study of the bidirectional association between pain and depressive symptoms in patients with psoriatic arthritis. Arthritis Care Res (Hoboken) 2012;64(5):758–765. 22231988 10.1002/acr.21602

[36] Brown GK . A causal analysis of chronic pain and depression. J Abnorm Psychol 1990;99(2):127–137. 2348006 10.1037//0021-843x.99.2.127

[37] Smedstad LM , Vaglum P , Moum T , et al. The relationship between psychological distress and traditional clinical variables: a 2 year prospective study of 216 patients with early rheumatoid arthritis. Br J Rheumatol 1997;36(12):1304–1311. 9448592 10.1093/rheumatology/36.12.1304

[38] Odegård S , Finset A , Mowinckel P , et al. Pain and psychological health status over a 10‐year period in patients with recent onset rheumatoid arthritis. Ann Rheum Dis 2007;66(9):1195–1201. 17392351 10.1136/ard.2006.064287 PMC1955161

[39] Hawley DJ , Wolfe F . Anxiety and depression in patients with rheumatoid arthritis: a prospective study of 400 patients. J Rheumatol 1988;15(6):932–941. 3418643

[40] International Association for the Study of Pain . IASP Announces Revised Definition of Pain. Published July 16, 2020. Accessed July 1, 2025. https://www.iasp-pain.org/publications/iasp-news/iasp-announces-revised-definition-of-pain/

[41] Raja SN , Carr DB , Cohen M , et al. The revised International Association for the Study of Pain definition of pain: concepts, challenges, and compromises. Pain 2020;161(9):1976–1982. 32694387 10.1097/j.pain.0000000000001939 PMC7680716

[42] International Association for the Study of Pain . Terminology. Accessed August 1, 2024. https://www.iasp-pain.org/resources/terminology/?ItemNumber=1698#Pain

[43] Al Mohamad F , Rios Rodriguez V , Haibel H , et al. Association of nociplastic and neuropathic pain components with the presence of residual symptoms in patients with axial spondyloarthritis receiving biological disease‐modifying antirheumatic drugs. RMD Open 2024;10(1):e004009. 38360039 10.1136/rmdopen-2023-004009 PMC10875534

[44] Khot S , Tackley G , Choy E . How to distinguish non‐inflammatory from inflammatory pain in RA? Curr Rheumatol Rep 2024;26(12):403–413. 39120749 10.1007/s11926-024-01159-4 PMC11527911

[45] Rutter‐Locher Z , Arumalla N , Norton S , et al. A systematic review and meta‐analysis of questionnaires to screen for pain sensitisation and neuropathic like pain in inflammatory arthritis. Semin Arthritis Rheum 2023;61:152207. 37163841 10.1016/j.semarthrit.2023.152207

[46] Duffield SJ , Miller N , Zhao S , et al. Concomitant fibromyalgia complicating chronic inflammatory arthritis: a systematic review and meta‐analysis. Rheumatology (Oxford) 2018;57(8):1453–1460. 29788461 10.1093/rheumatology/key112 PMC6055651

[47] Freynhagen R , Parada HA , Calderon‐Ospina CA , et al. Current understanding of the mixed pain concept: a brief narrative review. Curr Med Res Opin 2019;35(6):1011–1018. 30479161 10.1080/03007995.2018.1552042

[48] Jesulola E , Micalos P , Baguley IJ . Understanding the pathophysiology of depression: from monoamines to the neurogenesis hypothesis model ‐ are we there yet? Behav Brain Res 2018;341:79–90. 29284108 10.1016/j.bbr.2017.12.025

[49] Ionescu CE , Popescu CC , Agache M , et al. Depression in rheumatoid arthritis: a narrative review‐diagnostic challenges, pathogenic mechanisms and effects. Medicina (Kaunas) 2022;58(11):1637. 36422176 10.3390/medicina58111637 PMC9696661

[50] Brown GK , Nicassio PM , Wallston KA . Pain coping strategies and depression in rheumatoid arthritis. J Consult Clin Psychol 1989;57(5):652–657. 2794186 10.1037//0022-006x.57.5.652

[51] Chen MH , Sun CK , Lin IM , et al. Size reduction of the right amygdala in chronic pain patients with emotional stress: a systematic review and meta‐analysis. Pain Med 2023;24(5):556–565. 36308460 10.1093/pm/pnac162

[52] Zheng CJ , Van Drunen S , Egorova‐Brumley N . Neural correlates of co‐occurring pain and depression: an activation‐likelihood estimation (ALE) meta‐analysis and systematic review. Transl Psychiatry 2022;12(1):196. 35545623 10.1038/s41398-022-01949-3 PMC9095719

[53] Ma T , Ji YY , Yan LF , et al. Gray matter volume abnormality in chronic pain patients with depressive symptoms: a systemic review and meta‐analysis of voxel‐based morphometry studies. Front Neurosci 2022;16:826759. 35733934 10.3389/fnins.2022.826759 PMC9207409

[54] Malfliet A , Coppieters I , Van Wilgen P , et al. Brain changes associated with cognitive and emotional factors in chronic pain: a systematic review. Eur J Pain 2017;21(5):769–786. 28146315 10.1002/ejp.1003

[55] Thompson T , Correll CU , Gallop K , et al. Is pain perception altered in people with depression? A systematic review and meta‐analysis of experimental pain research. J Pain 2016;17(12):1257–1272. 27589910 10.1016/j.jpain.2016.08.007

[56] Karimi R , Mallah N , Scherer R , et al. Sleep quality as a mediator of the relation between depression and chronic pain: a systematic review and meta‐analysis. Br J Anaesth 2023;130(6):747–762. 37059623 10.1016/j.bja.2023.02.036

[57] Antoniou G , Lambourg E , Steele JD , et al. The effect of adverse childhood experiences on chronic pain and major depression in adulthood: a systematic review and meta‐analysis. Br J Anaesth 2023;130(6):729–746. 37087334 10.1016/j.bja.2023.03.008 PMC10251130

[58] Khan WU , Michelini G , Battaglia M . Twin studies of the covariation of pain with depression and anxiety: a systematic review and re‐evaluation of critical needs. Neurosci Biobehav Rev 2020;111:135–148. 31954722 10.1016/j.neubiorev.2020.01.015

[59] Dresler T , Caratozzolo S , Guldolf K , et al; European Headache Federation School of Advanced Studies (EHF‐SAS). Understanding the nature of psychiatric comorbidity in migraine: a systematic review focused on interactions and treatment implications. J Headache Pain 2019;20(1):51. 31072313 10.1186/s10194-019-0988-x PMC6734261

[60] Martucci KT , Ng P , Mackey S . Neuroimaging chronic pain: what have we learned and where are we going? Future Neurol 2014;9(6):615–626. 28163658 10.2217/FNL.14.57 PMC5289824

[61] Kirby LAJ , Robinson JL . Affective mapping: an activation likelihood estimation (ALE) meta‐analysis. Brain Cogn 2017;118:137–148. 26074298 10.1016/j.bandc.2015.04.006

[62] Fraenkel L , Bathon JM , England BR , et al. 2021 American College of Rheumatology guideline for the treatment of rheumatoid arthritis. Arthritis Rheumatol 2021;73(7):1108–1123. 34101376 10.1002/art.41752

[63] National Institute for Health and Care Excellence . Rheumatoid arthritis in adults: management. Updated October 12, 2020. Accessed November 11, 2025. https://www.nice.org.uk/guidance/ng100

[64] National Institute for Health and Care Excellence . Spondyloarthritis in over 16s: diagnosis and management. Updated June 2, 2017. Accessed November 1, 2025. https://www.nice.org.uk/guidance/ng65 28350428

[65] Singh JA , Guyatt G , Ogdie A , et al. Special article: 2018 American College of Rheumatology/National Psoriasis Foundation guideline for the treatment of psoriatic arthritis. Arthritis Rheumatol 2019;71(1):5–32. 30499246 10.1002/art.40726 PMC8218333

[66] Ward MM , Deodhar A , Gensler LS , et al. 2019 Update of the American College of Rheumatology/Spondylitis Association of America/Spondyloarthritis Research and Treatment Network recommendations for the treatment of ankylosing spondylitis and nonradiographic axial spondyloarthritis. Arthritis Rheumatol 2019;71(10):1599–1613. 31436036 10.1002/art.41042 PMC6764882

[67] Geenen R , Overman CL , Christensen R , et al. EULAR recommendations for the health professional's approach to pain management in inflammatory arthritis and osteoarthritis. Ann Rheum Dis 2018;77(6):797–807. 29724726 10.1136/annrheumdis-2017-212662

[68] Scott IC , Babatunde O , Barker C , et al. Pain management in people with inflammatory arthritis: British Society for Rheumatology guideline scope. Rheumatol Adv Pract 2024;8(4):rkae128. 39563967 10.1093/rap/rkae128 PMC11573413

[69] Nikiphorou E , Santos EJF , Marques A , et al. 2021 EULAR recommendations for the implementation of self‐management strategies in patients with inflammatory arthritis. Ann Rheum Dis 2021;80(10):1278–1285. 33962964 10.1136/annrheumdis-2021-220249 PMC8458093

[70] National Institute for Health and Care Excellence . Depression in adults with a chronic physical health problem: recognition and management. Published October 28, 2009. Accessed November 1, 2025. https://www.nice.org.uk/guidance/cg91 39808015

[71] Barry MJ , Nicholson WK , Silverstein M , et al; US Preventive Services Task Force. Screening for depression and suicide risk in adults: US Preventive Services Task Force recommendation statement. JAMA 2023;329(23):2057–2067. 37338872 10.1001/jama.2023.9297

[72] Barnabe C , Wattiaux A , Petkovic J , et al. Validation studies of rheumatoid arthritis patient‐reported outcome measures in populations at risk for inequity: a systematic review and analysis using the OMERACT summary of measurement properties equity table. Semin Arthritis Rheum 2022;55:152029. 35640489 10.1016/j.semarthrit.2022.152029

[73] Englbrecht M , Tarner IH , van der Heijde DM , et al. Measuring pain and efficacy of pain treatment in inflammatory arthritis: a systematic literature review. J Rheumatol Suppl 2012;90:3–10. 22942322 10.3899/jrheum.120335

[74] Victoria MA , Lucio VR , Cristina HD . Patient self‐reported instruments for assessing symptoms in rheumatoid arthritis. Rheumatol Int 2023;43(10):1781–1790. 37322354 10.1007/s00296-023-05355-w

[75] Ortega‐Avila AB , Ramos‐Petersen L , Cervera‐Garvi P , et al. Systematic review of the psychometric properties of patient‐reported outcome measures for rheumatoid arthritis in the foot and ankle. Clin Rehabil 2019;33(11):1788–1799. 31291785 10.1177/0269215519862328

[76] van der Leeden M , Steultjens MPM , Terwee CB , et al. A systematic review of instruments measuring foot function, foot pain, and foot‐related disability in patients with rheumatoid arthritis. Arthritis Rheum 2008;59(9):1257–1269. 18759256 10.1002/art.24016

[77] Dickens C , McGowan L , Clark‐Carter D , et al. Depression in rheumatoid arthritis: a systematic review of the literature with meta‐analysis. Psychosom Med 2002;64(1):52–60. 11818586 10.1097/00006842-200201000-00008

[78] Teuwen MMH , Knaapen IRE , Vliet Vlieland TPM , et al. The use of PROMIS measures in clinical studies in patients with inflammatory arthritis: a systematic review. Qual Life Res 2023;32(10):2731–2749. 37103773 10.1007/s11136-023-03422-0 PMC10474175

[79] Hansen CW , Esbensen BA , de Thurah A , et al. Outcome measures in rheumatology applied in self‐management interventions targeting people with inflammatory arthritis: a systematic review of outcome domains and measurement instruments. Semin Arthritis Rheum 2022;54:151995. 35397237 10.1016/j.semarthrit.2022.151995

[80] Küçükdeveci AA , Elhan AH , Erdoğan BD , et al. Use and detailed metric properties of patient‐reported outcome measures for rheumatoid arthritis: a systematic review covering two decades. RMD Open 2021;7(2):e001707. 34376556 10.1136/rmdopen-2021-001707 PMC8356163

[81] Minnock P , McKee G , Kelly A , et al. Nursing sensitive outcomes in patients with rheumatoid arthritis: a systematic literature review. Int J Nurs Stud 2018;77:115–129. 29080437 10.1016/j.ijnurstu.2017.09.005

[82] Tang AC , Kim H , Crawford B , et al. The use of patient reported outcome measures for rheumatoid arthritis in Japan: a systematic literature review. Open Rheumatol J 2017;11(1):43–52. 28553419 10.2174/1874312901711010043 PMC5427692

[83] Kilic L , Erden A , Bingham CO III , et al. The reporting of patient‐reported outcomes in studies of patients with rheumatoid arthritis: a systematic review of 250 articles. J Rheumatol 2016;43(7):1300–1305. 27084908 10.3899/jrheum.151177

[84] Cox N , Kettle C , Wang H , et al. E041 Feasibility and acceptability of electronic patient reported outcome measures in the routine care of people with inflammatory arthritis: Haywood Arthritis Portal study. Rheumatology 2024;63(Supplement_1):keae163.269.

[85] Scott IC , Whittle R , Bailey J , et al. Rheumatoid arthritis, psoriatic arthritis, and axial spondyloarthritis epidemiology in England from 2004 to 2020: an observational study using primary care electronic health record data. Lancet Reg Health Eur 2022;23:100519. 36246147 10.1016/j.lanepe.2022.100519 PMC9557034

[86] Office for National Statistics . Internet access ‐ households and individuals QMI. Updated August 8, 2019. Accessed November 1, 2025. https://www.ons.gov.uk/peoplepopulationandcommunity/householdcharacteristics/homeinternetandsocialmediausage/methodologies/internetaccesshouseholdsandindividualsqmi

[87] Lloyds Bank . UK Consumer Digital Index. Accessed November 1, 2025. https://www.lloydsbank.com/banking‐with‐us/whats‐happening/consumer‐digital‐index.html

