# Supplementary figures and images for: Longitudinal Relationship Between Pain and Depression in People With Inflammatory Arthritis: A Narrative Review
Source: Arthritis Care Res (Hoboken). 2025 May 8;78(1):15–30. doi: 10.1002/acr.25532 (PMC12826089; doi:10.1002/acr.25532)

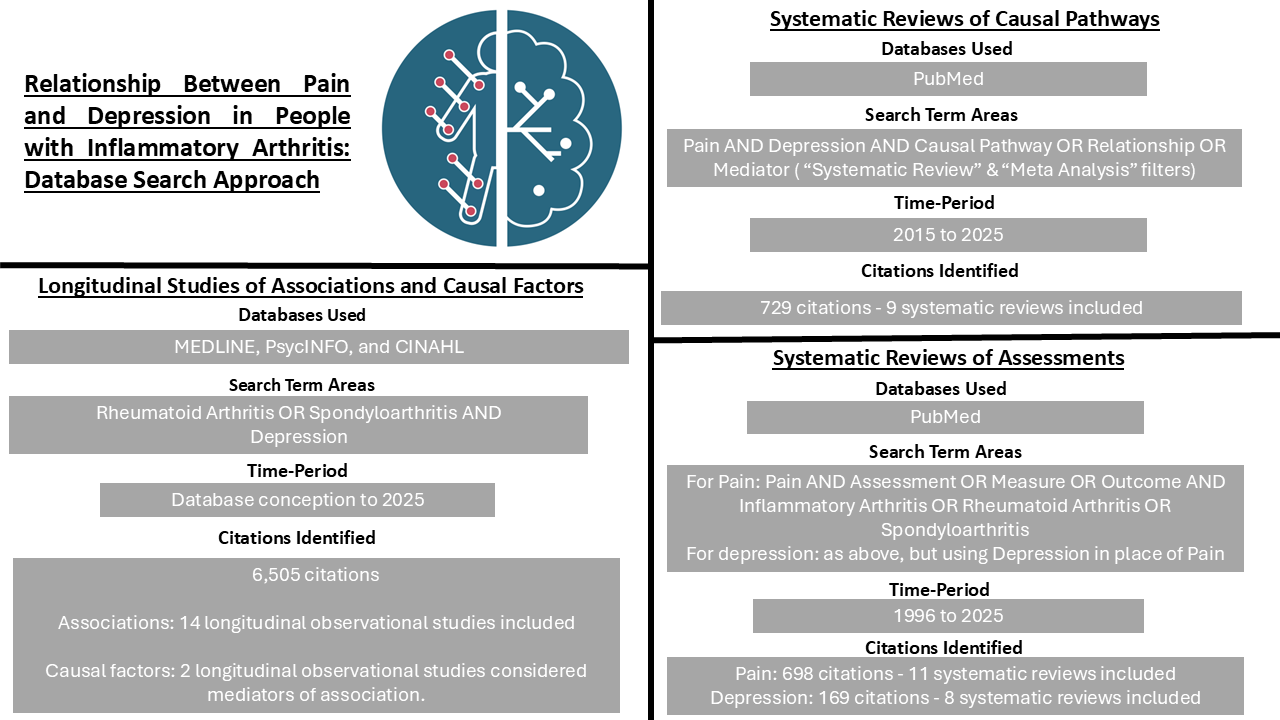

Supplement: Supplementary file 2 — Supplementary Figure 1: [file ACR-78-15-s002.tiff]
